# Symbiotic N fixation is sufficient to support net aboveground biomass accumulation in a humid tropical forest

**DOI:** 10.1038/s41598-019-43962-5

**Published:** 2019-05-20

**Authors:** E. N. J. Brookshire, Nina Wurzburger, Bryce Currey, Duncan N. L. Menge, Michael P. Oatham, Carlton Roberts

**Affiliations:** 1 Department of Land Resources and Environmental Sciences, Montana State University, Bozeman, USA; 2 Odum School of Ecology, University of Georgia, Athens, USA; 3 Department of Ecology, Evolution, and Environmental Biology, Columbia University, New York, USA; 4 Department of Life Sciences, University of the West Indies, St. Augustine, St. Augustine, Trinidad and Tobago; 5 Trinidad Ministry of Agriculture, Land and Fisheries, Forestry Division, Point Fortin, Trinidad and Tobago

**Keywords:** Element cycles, Ecosystem ecology

## Abstract

Symbiotic nitrogen (N) fixation has been shown to support carbon storage in young regenerating tropical forests, but N-fixing trees can also be strong competitors with non-fixing trees, making it unclear which mechanism drives long term patterns in biomass accretion. Many tropical forests have excess N, but factors such as rising atmospheric CO_2_ or selective cutting practices might induce additional N demand. Here we combine decades of stem inventory data, *in-situ* measures of symbiotic N fixation, and simulations of N demand to evaluate demographic and biogeochemical controls on biomass dynamics in legume-rich lowland forests of Trinidad. We document sustained net biomass accumulation and high rates of N fixation in these forests, regardless of the timing of selective timber harvests, including an old growth stand. The biomass accumulation was explained by growth of non-fixing trees, not N-fixing trees, but the total amount of symbiotic N fixation was sufficient to account for most of net above ground N demands, suggesting that N-fixers could contribute to the long-term C sink in these forests via fertilizing non-fixers.

## Introduction

Humid tropical forests are linchpins in the global carbon (C) cycle, removing more atmospheric CO_2_ annually than any other terrestrial biome^1^. In many tropical forests worldwide, above ground biomass (AGB) has increased over the last few decades^2–4^ and has contributed substantially to the global land CO_2_ sink^5^. However, the future of this C sink is highly uncertain^6^ in large part because the extent to which soil nutrients constrain this C sink remains unclear^7–9^.

Net biomass accumulation in mature tropical forests requires large quantities of nitrogen (N) and other nutrients. One group of plants, symbiotic nitrogen (N)-fixers, is particularly intriguing in this respect because it can access an inexhaustible pool of N (atmospheric N_2_ gas). Indeed, symbiotic biological N fixation (BNF) has been shown to support AGB accumulation in tropical plantations^10^ and young (~2–40 years old) secondary forests^11,12^. However, the extent to which this is true for older (i.e., “mature”; >100 years^13^) forests is unclear^14,15^. Furthermore, in many tropical regions, otherwise “mature” forests that have never been cleared or converted to pasture/agriculture are nevertheless subject to selective cutting practices. Such small-scale disturbances can represent a large net source of CO_2_^16^ but also result in vigorous regrowth^17^.

There are a couple reasons to suspect that symbiotic N fixation might not be necessary for AGB accumulation in mature tropical forests. First, many mature tropical forests are often considered to be limited by rock-derived nutrients (e.g., P, Ca, K) rather than N^14,18^, though both have been found to limit some aspect of primary production in fertilization studies^19,20^ and observational studies across mature forests have found positive correlations between AGB and both soil N^21^ and soil P^22^. Second, many tropical legumes are thought to be facultative N-fixers (BNF is down-regulated under N-rich conditions^14,23–25^) and many mature tropical forests are N-rich^26,27^ suggesting that little legume BNF occurs in mature forests. However, only a handful of studies have quantified symbiotic BNF in mature tropical forests globally^12,25,28–34^, making generalizations difficult. Although most of these studies support the idea that BNF is lower in mature than aggrading forests, they raise the critical question as to whether BNF is sufficient to sustain net biomass growth. While the majority of the N required to support annual net primary production in mature tropical forests is supplied by internal recycling^15,27,35^, *net* biomass accumulation eventually requires *new* N given the high N loss rates characteristic of these ecosystems^26,27,36,37^. Understanding the extent to which BNF can support this net growth, and for how long, is critical for predicting the future of the tropical C sink.

N-fixer-driven C storage could stem from one of two mechanisms. N-fixers may influence forest-wide biomass dynamics directly via their demographic rates, or they may supply new N to their neighboring trees via turnover and decomposition of their tissues. For N-fixers to drive net AGB accumulation directly, the sum of biomass gains from stem diameter growth and recruitment of new stems must be greater than turnover (mortality) and this net positive AGB change must be greater than that of non-fixers. Both Batterman *et al*.^12^ and Menge and Chazdon^38^ found that N-fixing trees (in Panama and Costa Rica, respectively) possess direct demographic advantages (higher growth and survival) over neighboring non-fixing trees in early succession, but not in mature forests, suggesting AGB accumulation may not be higher with more N-fixers due to demographics alone. However, mature forests at both sites sustained BNF at levels >2 kg N ha^−1^ yr^−1^ and it is unclear whether these forests were gaining AGB and if this N input was sufficient to support it. Further, it is possible that N-fixers could negatively influence forest growth if they effectively compete for resources with other trees. Indeed, the one study that has explicitly examined the direct and indirect effects of N-fixers, found that the demographic advantages of fixers were offset by strong competitive effects on their neighbors, resulting in a net negative effect on forest growth early in succession, but less so later in succession^39^. These observations identify major uncertainties in our understanding of the net effects of N-fixers on net AGB accumulation in mature tropical forests.

To disentangle direct (demographic) and indirect (N supply) effects of N-fixing trees on forest biomass accretion requires long-term stem inventories at sites where BNF has been directly quantified, yet these data are rare. Here, we make use of a unique >30 year record of tree stem dynamics in lowland humid forests of the Victoria-Mayaro Forest Reserve (VMFR) in Trinidad to ask how N-fixers influence net AGB accumulation in lowland tropical forests that have never been clear cut or converted to pasture or agriculture. Our plots include old-growth forests and those subjected to a single event of selective logging (i.e., 2–4 trees ha^−1^ over the last 30 years; Supplementary Table S1). We combine stem data with field measures of BNF and soil chemistry and simple modeling of plant N demand to determine whether (1) forest AGB has changed directionally over time; (2) N-fixing trees contribute to long-term AGB dynamics directly via their demographic rates; (3) measured rates of BNF are sufficient to supply N demands required to sustain observed AGB change of N-fixers and non-fixers.

## Results

### Tree demographics and biomass dynamics

**Figure 1:**
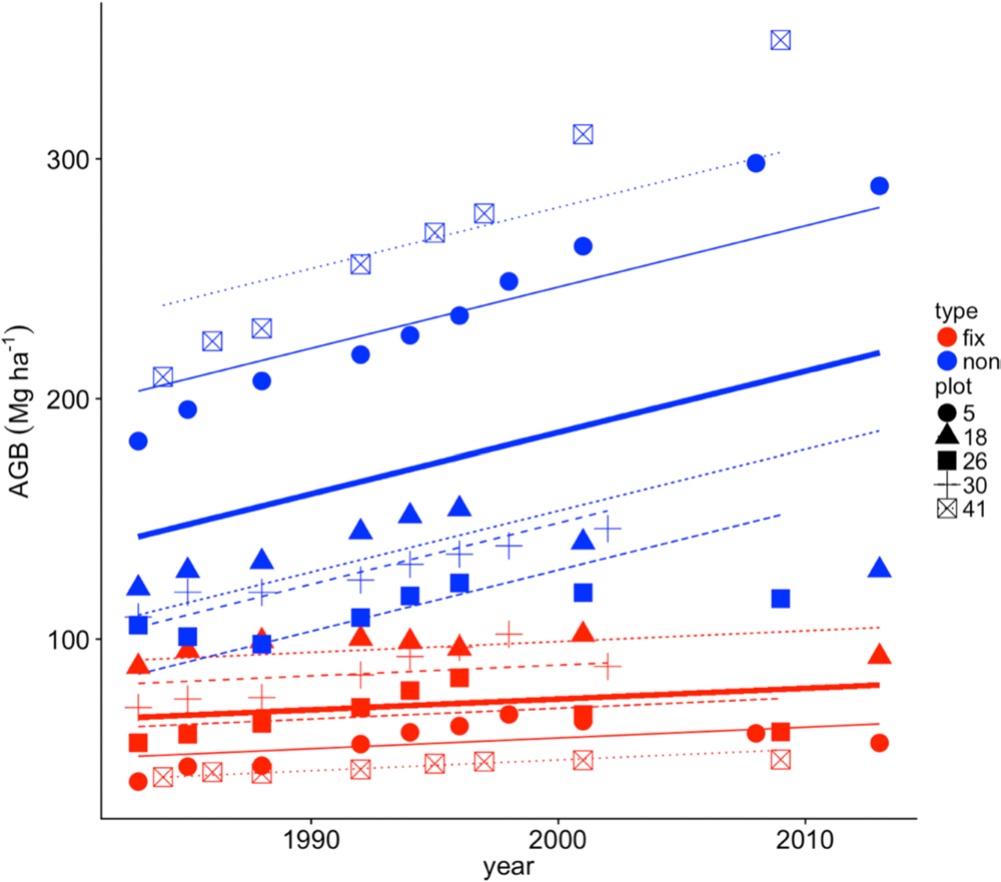
Aboveground biomass (AGB) for the five 1-ha stem inventory plots in the VMFR over time. Red points and lines are N-fixers and blue points and lines are non-fixers. Thick red and blue lines show the average change in AGB across plots, while thin lines and different symbols represent individual plots. All slopes are fixed effects from a linear mixed effects model with a significant interaction between tree type (“type”) and time and a random effect of tree type nested within plot. Plots were either old-growth and never cut (Plot 41) or selectively harvested in years 1981 (Plot 5), 2000 (Plot 18), 2005 (Plot 26) or 2012 (Plot 30).

VMFR forests display a wide range of mean long-term total AGB across plots (162–312 Mg ha^−1^). Non-fixing trees contributed a larger portion of AGB than did N-fixing trees (*P* = 0.049; using tree “type” (i.e., N-fixer vs. non-fixer) as a fixed effect), and AGB increased over time for non-fixers only (tree type by year interaction in linear mixed effects model with plot identity as a random effect, *P* < 0.001; ${R}_{m}^{2}$ = 0.58, ${R}_{c}^{2}$ = 0.96; Fig. 1). Overall, AGB of non-fixers increased by an average of 35% (77 Mg ha^−1^) across plots over the ~30 years of measurement. In contrast, N-fixer AGB remained much more constant across plots over time (Fig. 1, Supplementary Table S2). Increasing AGB among non-fixers and stable AGB among N-fixers resulted in long term increases in total AGB in all plots, yielding a mean AGB of 276 Mg ha^−1^ (178–400 Mg ha^−1^) in the final census. While we observed decreases in AGB (8–10 Mg ha^−1^; 3–5% of total AGB) following harvest in the two plots for which we have pre- and post-harvest data (Plots 18 and 26), this represented only 3–5% of total AGB, indicating that other factors are more important in governing relative AGB increases and the proportion of N-fixers and non-fixers across plots. In fact, AGB accumulated most strongly over time in the old-growth plot (Plot 41), AGB increased at similar relative rates across all plots prior to cutting and the relative order of AGB among plots was maintained over time regardless of harvest events.

**Figure 2:**
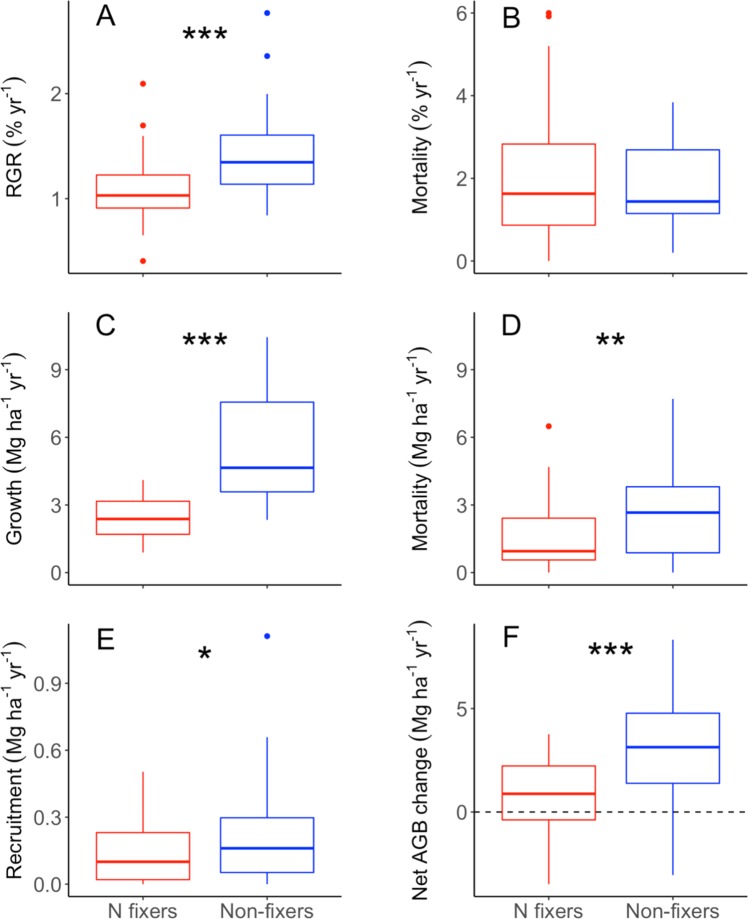
Long-term demographic rates and biomass change for N-fixers (red) and non-fixers (blue box plots) across all plots. In the box plots, the thick line is the median, the edges of the box are 25% and 75% percentiles, the whiskers representing 1.5 times inter quartile range, and dots represent outliers. Asterisks indicate level of significance for tests of differences between N-fixers and non-fixers (*P < 0.05, **P < 0.01, ***P < 0.001).

We next considered how these long-term patterns in forest AGB emerged from differences in demographic rates between N-fixing and non-fixing trees. For all demographic models, we found no significant interactions between tree type and year (Supplementary Table S2). Individual-level relative growth rates (RGR) was higher for non-fixing than N-fixing stems (*P* < 0.001; Fig. 2A) and RGR for both types decreased over time (~20%, *P* < 0.001, ${R}_{m}^{2}$ = 0.32, ${R}_{c}^{2}$ = 0.55). Similarly, annual biomass growth was consistently higher for non-fixing than N-fixing trees (*P* < 0.001; Fig. 2C) and decreased over time (*P* = 0.29, ${R}_{m}^{2}$ = 0.44, ${R}_{c}^{2}$ = 0.86). Recruitment of new stems was slightly higher for non-fixing than N-fixing trees (*P* = 0.049; Fig. 2E) did not vary across time (*P* = 0.25) and did not depend on plot identity (${R}_{m}^{2}$ = ${R}_{c}^{2}$ = 0.07). Recruitment of N-fixers and non-fixers combined accounted for <5% of total biomass gains (i.e., most biomass accumulation was driven by annual diameter growth of existing trees; Fig. 2C).

In contrast to long-term patterns in stem biomass growth, annual stem mortality rates (%) did not differ between N-fixing and non-fixing trees (*P* = 0.22; Fig. 2B) and increased ~2 to 3% over time (with time since harvest as a random effect, *P* = 0.0035; ${R}_{m}^{2}$ = 0.14, ${R}_{c}^{2}$ = 0.38; Supplementary Table S2). Because non-fixers comprise a larger fraction of total AGB, similar individual-level mortality rates translate to higher stand-level biomass turnover for non-fixers than N-fixers (*P* = 0.0029; Fig. 2d). Stand-level turnover increased over time for both fixers and non-fixers (*P* = 0.0192; ${R}_{m}^{2}$ = 0.19, ${R}_{c}^{2}$ = 0.28; Supplementary Table S2). While AGB decreased following selective harvest in two plots, mixed effects models using time since harvest as a fixed effect instead of year (i.e., Y ~ Type + Time, random = ~1| Plot) were not competitive candidate models (higher AIC) and indicted no effect on mortality rates or biomass turnover (*P* > 0.088).

Despite higher biomass turnover among non-fixers than N-fixers, non-fixers contributed significantly more to net AGB gains than N-fixers (*P*  < 0.001) over time (*P* = 0.0218, ${R}_{m}^{2}$ = 0.24, ${R}_{c}^{2}$ = 0.44; Fig. 2f). Total AGB change across plots and over time was positively associated with annual growth of non-fixers and negatively associated with mortality of both N-fixers and non-fixers (Supplementary Fig. S1).

### Nodulation and N fixation

**Figure 3:**
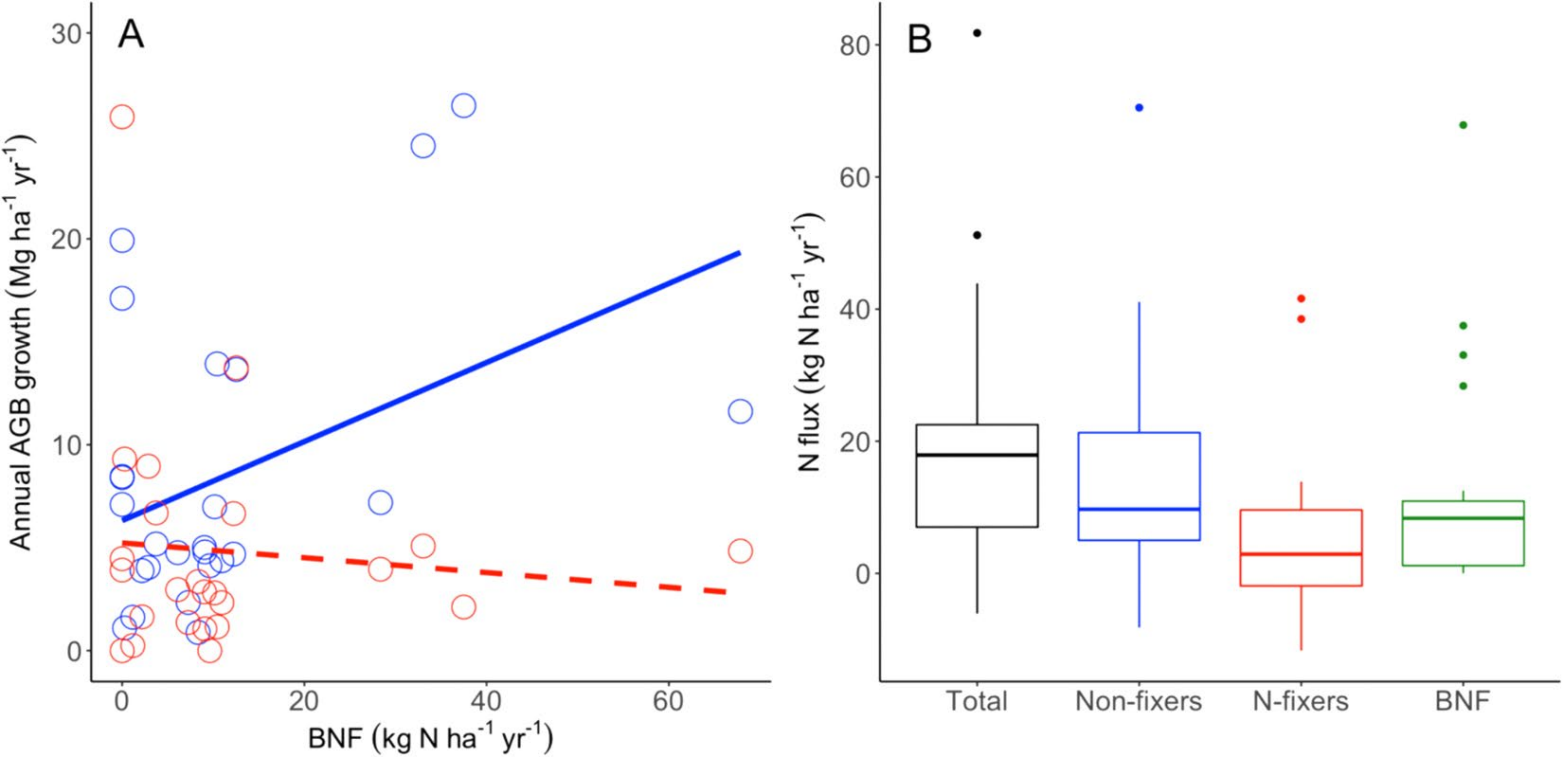
Observed patterns of tree growth, BNF and simulated N demand. (**A**) Observed biomass growth for N-fixers (red) and non-fixers (blue) at the subplot scale for the most recent census interval versus BNF measured in 2015. Lines show results for fixed effects in a linear mixed effects model with a significant interaction between tree type and BNF and a random effect of plot. (**B**) Simulated N demands for long-term net incremental AGB change for all trees, non-fixers and N-fixers compared to estimated rates of BNF across all sub plots in which BNF was measured (*n* = 25). Box plots show the median, whiskers and outliers.

We quantified legume nodulation and estimated BNF within five randomly selected 20 × 20 m subplots within each of our five 1-ha plots. We found high levels of nodulation, nodule biomass and N fixation across these forests. Across our 25 subplots, we documented 84 potential N-fixing trees (average of 3.4 stems per 400 m^2^ subplot). We found nodules near 65% of these 84 trees (Table 1). *Pentaclethra* accounted for 83% of these nodulated stems, with *Inga* spp., and *Swartzia* spp. and *Lonchocarpus punctatus* making up the remainder. Estimated subplot-level BNF varied widely across subplots (range = 0–67.8 kg ha^−1^ yr^−1^). While average (*n* = 5) plot-level nodule biomass (0.7–5.1 g m^−2^) and BNF (mean = 11.3 kg N ha^−1^ yr^−1^; range = 4.1–24.2 kg N ha^−1^ yr^−1^; Table 1) also varied considerably, plot identity had no significant random effect on subplot-level BNF for any model. Also, we found no relationship between nodule biomass or BNF with extractable soil inorganic N at the individual tree or subplot scale (Supplementary Fig. S2). Across all biomass, demographic and biogeochemical measures, the only significant and positive associations with BNF were for subplot-level total AGB (*P* = 0.026), non-fixer AGB (*P* = 0.027) and, most strongly, non-fixer annual growth rate (*P* = 0.011; Fig. 3A; Supplementary Table S3), although the relationship is still noisy.

**Table 1:** Relative abundance of N-fixing trees, percent of N fixers with nodules, nodule biomass, symbiotic biological nitrogen fixation (BNF) and extractable inorganic N in VMFR forests.

Plot	N-fixers (% of stems)	Nodulation (% of fixers)	Nodule biomass (g m^−2^)	BNF (kg N ha^−1^ yr^−1^)	Inorganic N (μg N g dry soil^−1^)
5	38.2 (9.5)	55.3 (6.2)	1.4 (0.7)	9.7 (6.2)	8.2 (1.6)
18	32.6 (5.7)	56.6 (19.4)	1.3 (1.2)	4.9 (2.5)	8.3 (1.7)
26	46.0 (7.8)	66.6 (21.1)	0.7 (0.5)	4.1 (2.0)	11.6 (5)
30	52.7 (3.5)	90.0 (10.0)	5.1 (2.9)	24.2 (11.6)	12.1 (2.7)
41	18.4 (6.2)	73.3 (19.4)	2.7 (1.1)	13.5 (6.3)	5.4 (1.1)

All values are arithmetic means (± SE) of 20 × 20 m subplots (*n* = 5 per 1-ha plot).

### Nitrogen demand

We assume that most of the long-term annual N demand for aboveground tree growth  was met by internal recycling and so we focused on the new N required to sustain *net* incremental AGB change. We estimate that the mean long-term incremental N demand was 5.4 kg N ha^−1^ yr^−1^ (2.5 and 97.5% quantiles [q2.5–97.5] = −57.2–47.2 kg N ha^−1^ yr^−1^) for N-fixers and 11.3 kg N ha^−1^ yr^−1^(−63.3–66.5 kg N ha^−1^ yr^−1^) for non-fixers (Fig. 3B). Applying our estimates of BNF from 2015 to the most recent census interval (which showed large net AGB accumulation), we calculate that BNF could have supplied on average ~55% of new N demand by N-fixers and ~24% of total new N demands over the last decade. If BNF rates over the past three decades were similar to the contemporary rate we measured, they would have supplied up to 200% of new N demands for N-fixers and 68% of forest-wide new N demands over those last three decades. Assuming this new N was entrained into the ecosystem-level N cycle, we estimate the potential average above ground C sink supported by BNF to be ~1.3–1.8 Mg C ha^−1^ yr^−1^.

## Discussion

Our examination of long-term stem dynamics of N-fixing and non-fixing trees points to sustained net aboveground C accumulation in these tropical forests over the last three decades. The aboveground C accumulation came primarily from non-fixers, which were more abundant and had higher relative growth rates than N-fixers. Although N-fixers did not contribute directly to aboveground C accumulation via their own biomass, their indirect facilitative effects could be substantial given that we documented high levels of nodulation and BNF rates. These contemporary BNF rates are a significant fraction of external N demands for aboveground biomass accumulation over the past few decades, so our study supports the idea that BNF may help sustain net C sequestration in these humid tropical forests.

Large increases in AGB in VMFR forests, including within an old-growth stand that has not sustained any logging, are broadly consistent with patterns observed across Amazonian mature forests^6^. Our VMFR forest plots display long-term increases in AGB at rates similar to those documented for secondary forests^40,41^, raising the question as to what triggered and sustained this growth. Unlike observations from many second-growth forests, annual diameter-based biomass growth has remained relatively stable over the period of record. Thus, AGB has increased over time because biomass growth, while not changing directionally over time, has been higher than biomass mortality. While some plots in the VMFR have been subject to selective harvest practices, none of our forests have ever been clear-cut, burned, or used for agriculture or livestock grazing. Cutting practices in the VMFR have been monitored since the early 1980’s and we did not detect an effect of selective harvest on any of the processes we analyzed. Further, these forests were studied in the early 1940’s by Beard^42,43^ and comparison of current species composition and diameter class distributions indicate little change since that time. Interestingly, all plots show similar slopes of AGB change over time regardless of selective harvest history (including our uncut old-growth plot), suggesting a common mechanism for triggering and sustaining AGB accumulation. Whether VMFR forests are recovering from past disturbance and/or are responding to atmosphere-climate change (e.g., CO_2_ fertilization) remains uncertain. Nevertheless, the range of current AGB across the landscape (178–400 Mg ha^−1^) is at the high end of mean AGB for “intact” forests in Central (146 Mg ha^−1^) and South America (266 Mg ha^−1^)^44^ indicating that VMFR forests display structural features common to mature humid tropical forests despite selective cutting practices.

Our analysis offers an interesting comparison to other tropical forests where stem demographics have been analyzed for N-fixers and non-fixers. For example, a Costa Rican forest displays a similarly high relative abundance of N-fixers as the VMFR, yet in Costa Rica, N-fixers (also dominated by *Pentaclethra*) grow faster and survive longer than non-fixers, particularly early in succession^38^. These findings suggest that high relative abundance of N-fixers could generate high AGB growth due to demographic differences alone. However, in these same Costa Rican forests, Taylor *et al*.^39^ reported that as the abundance of N-fixers increase, crowding (and presumably resource competition) by N-fixers suppresses the growth of neighboring N-fixing and non-fixing trees. This evidence for direct *inhibition* by N-fixers contrasts with general intuition and findings from elsewhere (e.g.,^11,12^) that BNF indirectly *facilitates* forest growth over the course of secondary succession. In contrast to these findings, Lai *et al*.^41^ recently found that the relative abundance of N-fixers had no net facilitative or inhibitive effect on total AGB recovery over the first ~30 years of secondary succession in lowland forests of Panama, though BNF was not quantified in this study. Combined, these contrasting observations suggest that the net effect of N-fixers on AGB accumulation in secondary forests depends not on the relative abundance of trees capable of N fixation per se but rather how much N they fix and how they interact with neighboring trees.

Recent studies in tropical forests of Panama have revealed the key importance of taxonomic identity to forest-wide BNF. Batterman *et al*.^12^ found that species contribution to BNF changed dynamically over the course of succession and Wurzburger and Hedin^30^ found taxonomic identity to be the strongest predictor of BNF in mature forests, with some species contributing little BNF across a range of resource (light and phosphorus) conditions while others were consistently fixing high levels of N, effectively functioning as “*superfixers*” across the landscape. In the case of the VMFR, *Pentaclethra* dominated BNF (80%) but also accounted for 84% of potentially N-fixing stems, suggesting that its relative importance to forest-wide BNF varied proportionally with its relative abundance.

How to best sample for nodules and estimate ecosystem-level symbiotic BNF in tropical forests is a subject of ongoing discussion^12,29,30,32,45^. We recognize that our systematic approach which restricted our survey to a fixed area around individual trees during a single wet season may have affected our estimates. However, given that we did not incorporate random sampling outside of this area but do account for the density of fixers in our areal estimates, we suspect it is more likely that we underestimated BNF. Further, our assumption of constant annual BNF rates is supported by a lack of wet-dry season variation in BNF in mature forests of Panama^24^ with a similar length dry season as ours. We document relatively high plot-level BNF rates (4.1–24.2 kg N ha^−1^ yr^−1^) compared to that reported for mature tropical forests worldwide^12,28–32,46^. In fact, BNF inputs to our old growth forests (13.5 kg N ha^−1^ yr^−1^) are similar to those measured in early successional *Pentaclethra*-rich forests in Costa Rica^34^. Yet, unlike reports from some secondary forest chronosequences, we find that BNF did not differ systematically with any environmental measure (including soil N availability) or selective harvest history.

Given the stability of N-fixer relative abundance in the VMFR over the last ~70 years or more^42^, these results suggest that N-fixers, and *Pentaclethra* in particular, could have supplied much of the N required to sustain historical levels of net AGB accumulation. However, there are several important considerations that may limit this inference. First, we did not measure or simulate below ground biomass N demand. Assuming that below ground tissues account for ~30–40% of total AGB^6,47^, inclusion of below ground N demand would reduce the percentage contribution of symbiotic BNF to total AGB accumulation. Second, our analysis did not account for other sources of external N inputs and did not consider the effects of ecosystem-level N losses on the relative importance of BNF. For example, N deposition to similar forests in Northern Trinidad is > 3 kg ha^−1^ yr^−1^ (ref.^37^) and asymbiotic N fixation can also bring significant quantities of N into mature tropical forests^34,48^. Further, mature tropical forests worldwide often display high levels of hydrologic and gaseous N loss^27^. Therefore, while our results point to a quantitatively important input from BNF in these forests, the extent to which BNF interacts with other external inputs and can redress external losses given high demand by accumulating non-fixer AGB is unclear.

Because symbiotic N fixation feeds N directly into host plants, this new N is only broadly available after it is entrained into the ecosystem N cycle via biomass turnover (including mortality). Remarkably, we find that BNF rates were up to three times greater than that required for long-term N-fixer net AGB accumulation and could supply nearly 60% of the new N required for total net AGB change in these forests. These findings offer evidence for a net positive effect of N-fixers on net biomass accumulation by non-fixers in these mature forests. In fact, our results showing that BNF was most positively related to non-fixer biomass growth suggests the intriguing possibility of feedbacks between N demand and supply in which N-fixers facilitate non-fixer AGB accumulation or, alternatively, BNF and non-fixer growth rates both increase in response to common (unmeasured) resource gradient.

Our results provide new quantitative insight into the role of BNF in the tropical forest C sink. Many tropical N-fixers are thought to down-regulate BNF under conditions of adequate soil N supply^14,24^. If this is the case, the persistence of N-fixers in our forests and their documented high levels of BNF suggests that tree growth may be N limited. Alternatively, our finding that nodulation and BNF did not vary with soil N availability suggests that BNF in these forests may be less facultative or is regulated by another resource. In fact, recent experimental work with *Pentaclethra* in Costa Rica indicates that N fixation by seedlings is more strongly regulated by light availability rather than by soil N availability^49^. In VMFR forests, annual relative stem growth and biomass productivity have not increased systematically over recent decades but have remained net positive and AGB has accumulated, a pattern consistent with CO_2_ fertilization or climate change and/or long-term recovery from disturbance. While we lack historical data on BNF, it is possible that individual-level BNF rates have changed given that the relative stem abundance and biomass of N-fixers have not changed appreciably while non-fixer AGB has steadily increased over time. The observation that these N-fixers are capable of supplying new N at levels sufficient to support N demands for long-term net AGB gain supports the idea that N-fixers can help sustain C sequestration in tropical forests.

## Methods

### Study site

We conducted our studies in lowland rainforests within the Victoria-Mayaro Forest Reserve (VMFR; 540 km^2^; 10°04′–10°18′N, 61°01′–61°18′W; elevation 25–80 masl) in southern Trinidad. Mean annual temperature is 27 °C and mean annual rainfall is 2200 mm distributed across distinct wet (June—December: 82% of annual rainfall) and dry (January—May) seasons. Trinidad is a continental island and was connected to mainland South America less than 10k years ago^50^. Soils in the VMFR are Ultisols and Vertisols^51,52^ derived from Middle and Lower Miocene sediments^53^. Our study forests are evergreen seasonal with a canopy height of ~35 m and contain 104 verified species of trees >20 cm in diameter at breast height (DBH). We note that some trees are identified to genus only and so total species richness is likely higher. Tree composition is dominated by *Pentaclethra macroloba* (hereafter “*Pentaclethra*”; ~30% of stems) and *Carapa guianensis* (~9%) with significant basal area contributions from *Pachira isignis* (~4%), *Trichilia pleeana* (~3%) and *Spondias mombin* (~2%)^42,54^. Though Trinidad lies outside of the hurricane belt, hurricanes do infrequently strike the island, the last of which was in 1933. While we lack information on any potential ecological effects of this event, Beard^42,43^ conducted extensive ecological studies in the VMFR in the early 1940’s and considered these “climax” forests lacking in any discernable large-scale disturbance.

We studied long-term stem data collected within an extensive network of 1-ha permanent sampling plots (hereafter “plots”; each divided into 25 20 × 20 m subplots) in the VMFR. Since 1983, the Trinidad Forestry Division has taxonomically identified and marked all individual trees >20 cm DBH (some species >10 cm) within each subplot every ~4 years and recorded growth, recruitment of new stems, and mortality^54^. These plots are managed as a periodic block system in which 2–4 trees are harvested per hectare in certain plots every ~30 years. The VMFR contains plots in which trees have been harvested once and plots in which trees have never been cut. None of the 1-ha plots have ever been clear-cut or converted to pasture or agriculture. In 2015, we randomly chose five 1-ha plots for investigation, four of which had been selectively harvested once since 1981 (Table S1). The minimum distance between plots was >2 km.

### Approach

We investigated forest dynamics and associated N demands in two ways: (1) we examined long term stem data and compared fixer versus non-fixer stem dynamics in our five 1-ha plots; (2). In 2015, we visited these sites and measured DBH, BNF and soil chemistry in a subset of 5 randomly selected subplots within each of our 5 plots and evaluated these against long-term stem dynamics and simulated N demands.

### Classification and allometry

For each plot, we taxonomically separated stem data into non-legumes, legumes that can nodulate and those that cannot (following Pons *et al*.^28^. Wood densities (*ρ*) were acquired from the Global Wood Density Database^55^. We used species-specific wood density when available (76% of stems) and otherwise used the mean genus-level (22%), mean family-level (0.5%) or, in a few cases, the South American tropical tree average wood density (1.5%). We corrected for measurement error for tree species that commonly buttress at or above DBH by 1) using diameters measured directly above the buttress apex in a 100-ha plot in the VMFR (United Nations Food and Agriculture Organization (FAO), 2016, *unpublished data*) and 2) comparing the diameter at both DBH and above the buttress for a set of individual stems (*n* = 13) in our five plots. Using these two methods, we checked the DBH of all stems in our plots that exceeded the geometric mean for commonly buttressing species from the FAO data set and applied a scaling relationship based on our field measures to estimate a corrected diameter *D*_*c*_ = 0.767* *DBH* + 1.115 (linear regression; *R*^2^ = 0.85, *P* < 0.001; Fig. S3). We used the allometric model of Alvarez *et al*.^56^; based on >600 trees ≥10 cm in DBH across multiple rainforests of Colombia) to estimate above ground biomass (AGB) for all trees: *ln*(*AGB*) = 1.662-1.114 *ln*(*D*) + 1.169 (*ln*(*D*))^2^ − 0.122 (*ln*(*D*))^3^ + 0.331 *ln*(*ρ*).

### Demographic and biomass dynamics

We followed similar protocols for the analysis of relative growth rates (RGR) following (ref.^57^) and AGB dynamics as (ref.^58^) and(ref.^6^). Relative growth rates were calculated as (*ln*[*d*_2_]-*ln*[*d*_1_])/(*t*_2_-*t*_1_) where *d*_2_ and *d*_1_ are stem DBH at the end (t_2_) and beginning (t_1_) of a given census interval. We checked stem data for inaccuracies including unrealistic growth (>5% yr^−1^), unrealistic shrinkage (<0% yr^−1^) and skipped censuses. In these cases, we estimated correct values via interpolation or by applying the species-specific mean growth rate for stems in the same plot belonging to the same diameter size class (<20 cm, 20–40 cm, 40–60 cm, >60 cm) following Brienen *et al*.^6^ Annual biomass growth was calculated from DBH-based allometric biomass change (DBH growth + recruitment) and mortality was based on a count of stems that went missing over a measurement interval. We corrected net biomass increment (which can be positive, zero, or negative) for the increasing probability that unmeasured recruits (>10 or 20 cm) grew and died with increasing census interval length following Brienen *et al*.^6^.

### BNF and soil chemistry

In the wet season (November) of 2015, we randomly selected five 20 × 20 m subplots within each of our five plots for measurement of stems, new recruits and BNF. To quantify BNF, we measured nodule biomass and conducted acetylene reduction assays (ARA) to determine specific nitrogenase activity at the individual tree level using methods of Wurzburger and Hedin^30^ with the following modifications. Within all 25 subplots, we sampled nodules by taking 9 randomly placed cores (4 cm diameter and 15 cm deep) within 2 m of the stem of every legume stem >10 cm DBH. For each legume, cores were composited and nodules were immediately incubated with 10% acetylene and gas samples were collected twice over 30 minutes and stored in glass vials for two weeks until analysis of ethylene concentration on a gas chromatograph (SRI 8610 C). All nodules were cleaned, dried to a constant mass at 60 °C and weighed. To scale up to tree-level N fixation rates, nodule biomass was multiplied by the specific nitrogenase activity of each tree. We assumed the theoretical C_2_H_2_:C_2_H_4_ to N_2_:2NH_4_ conversion ratio of 3. We summed tree-level areal (i.e., within 2 m of stems) nodule biomass and BNF within each subplot and divided this estimate by subplot area (400 m^2^) to scale to m^−2^ rates. Therefore, all nodule and BNF data are reported as per area of subplot or 1-ha plot rather than per area of tree canopy. For each tree, we homogenized the soil from the 9 cores and immediately extracted in the field for NO_3_-N and NH_4_-N in 2 N KCl. Extractions were periodically agitated for 4–6 hours, filtered and frozen until analysis using flow injection colorimetry on a QuikChem 8500 (Lachat Instruments).

### N demand simulations

We simulated aboveground N demands (*D*_*N*_) for fixers and non-fixers for all subplots in which we measured BNF by partitioning aboveground growth into wood and foliage and tissue-specific C:N ratios and applying these to our allometrically-derived AGB estimates through time following a similar approach as Brookshire *et al*.^27^.:

$${D}_{N}={\sum }_{i}\frac{\Delta AGB\ast {f}_{i}}{{S}_{i}}$$

Where Δ*AGB* represents observed net incremental growth (Mg ha^−1^ yr^−1^), *f*_*i*_ is the fraction of biomass allocated to tissue type *i* (wood or foliage) and *S*_*i*_ is the C:N ratio of the respective tissue. We assumed that C accounted for 50% of wood and leaf biomass for all N calculations following Brienen (2015). We restricted our analyses to measured components of aboveground biomass change and therefore ignored below ground biomass. We used published means and associated variance for the fractional contribution of wood and foliage to AGB and above-ground net primary production (ANPP) for humid tropical forests^26^. For wood stoichiometry, we applied published means and ranges for tropical legume and non-legume wood C:N ratios^59^. For foliar stoichiometry, we applied a range of C:N ratios for live leaves for N-fixing legumes and non-legumes^60,61^. To simulate new external N required to sustain net incremental AGB change, we applied the average fraction of AGB in foliage and wood in humid tropical forests and live wood and foliar C:N ratios for N-fixers and non-fixers. Using these calculations, we also compared the observed net aboveground C accumulation to that expected without external N supply from BNF assuming fixed stoichiometric ratios and that all fixed N is used to support aboveground growth. We conducted these simulations for the 25 subplots in which we measured BNF and stems in 2015 and for the full stem inventory record (*n* = 210 subplot census intervals across the five 1-ha plots over time). For all runs we evaluated effects of uncertainty for all parameters simultaneously using specific biomass allocation and stoichiometric data ranges and probability distributions (Supplementary Table S4) and simulating 10,000 times in a Monte Carlo framework.

### Statistics

We used linear mixed effects models fit by maximum likelihood to evaluate differences in AGB and demographic rates between tree type (N-fixer versus non-fixer) and how they change over time. We used the mid-point of each sampling interval for all demographic rate analyses. In all analyses, we first tested for significance of an interaction term between tree type and year and if this term was not significant we used an additive model. We accounted for plot-level variation in background conditions (e.g. soil resources and harvest history) in our statistical analyses by incorporating plot identity or tree type nested within plot as a random effect on the intercept. We also compared plot identity versus time since selective harvest as a random effect. For all model comparisons we selected the model with the lowest corrected (for small sample size versus number of parameters) Akaike Information Criterion (AICc) using an AICc difference (ΔAICc) between models >2 as a significance cutoff^62^. We determined the amount of variance explained by fixed (tree type and year) and random effects (plot) for all models by comparing marginal (${R}_{m}^{2}$; only fixed effects) and conditional *R*^2^ (${R}_{c}^{2}$; fixed and random effects)^63^. We used a similar approach to examine subplot-level variation in BNF by constructing a series of models using fixer and non-fixer biomass and demographics and soil extractable inorganic N as fixed effects and plot as random effect. All analyses were conducted in R v3.5.1^64^ using packages nlme^65^ and MuMIn^66^.

## Supplementary information

Symbiotic N fixation is sufficient to support net aboveground biomass accumulation in a humid tropical forest

## Data Availability

The datasets generated during and/or analysed during the current study are available from the corresponding author on reasonable request.
