# Supplementary material for: Symbiotic N fixation is sufficient to support net aboveground biomass accumulation in a humid tropical forest
Source: Sci Rep. 2019 May 20;9:7571. doi: 10.1038/s41598-019-43962-5 (PMC6527854; doi:10.1038/s41598-019-43962-5)
Supplement: Supplementary file 1 — Symbiotic N fixation is sufficient to support net aboveground biomass accumulation in a humid tropical forest [file 41598_2019_43962_MOESM1_ESM.pdf]

# Supplementary Information

**Symbiotic N fixation is sufficient to support net aboveground biomass accumulation in a humid tropical forest**

E.N.J Brookshire[1], Nina Wurzburger[2], Bryce Currey[1], Duncan N.L. Menge[3], Michael P. Oatham[4], Carlton Roberts[5]

1 Dept of Land Resources and Environmental Sciences, Montana State University; jbrookshire@montana.edu and brycecurrey93@gmail.com
2 Odum School of Ecology, University of Georgia;
ninawurz@uga.edu
3 Dept of Ecology, Evolution, and Environmental Biology, Columbia University; dm2972@columbia.edu
4 Dept of Life Sciences, University of the West Indies, St. Augustine;
Mike.oatham@sta.uwi.edu
5 Trinidad Ministry of Agriculture, Land and Fisheries, Forestry Division;
Carlton.roberts@yahoo.com

**Corresponding author: jbrookshire@montana.edu**

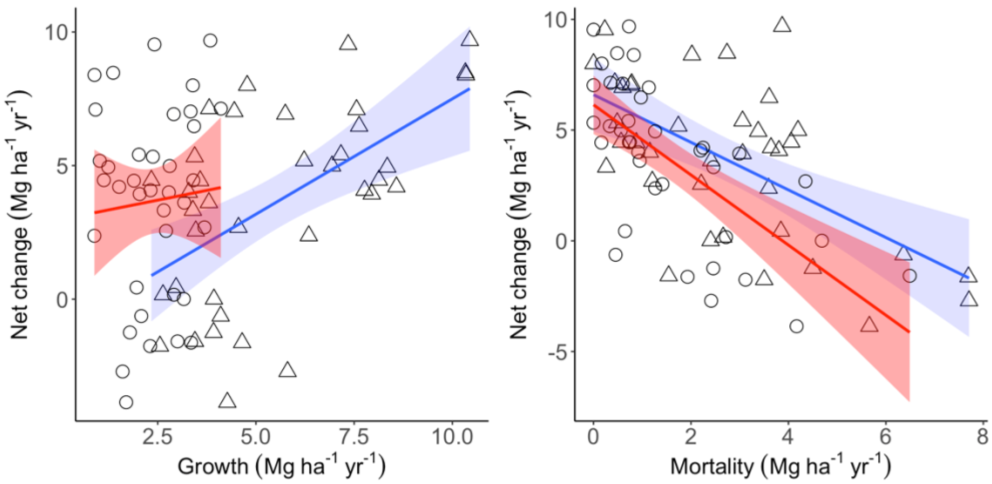


**Fig. S1.** Linear regressions showing effect of annual AGB growth and mortality on net incremental AGB change across five 1-ha VMFR forest plots. Each point represents one plot-census interval for N-fixers (circles and red lines and confidence 95% intervals) and non-fixers (triangles and blue lines and confidence intervals). Net biomass change across plots and over time was positively associated with annual growth of non-fixers ($R^2$= 0.30, $P$<0.001) and negatively associated with mortality of N-fixers ($R^2$= 0.42, $P$<0.0001) and non-fixers ($R^2$= 0.33, $P$<0.0001).

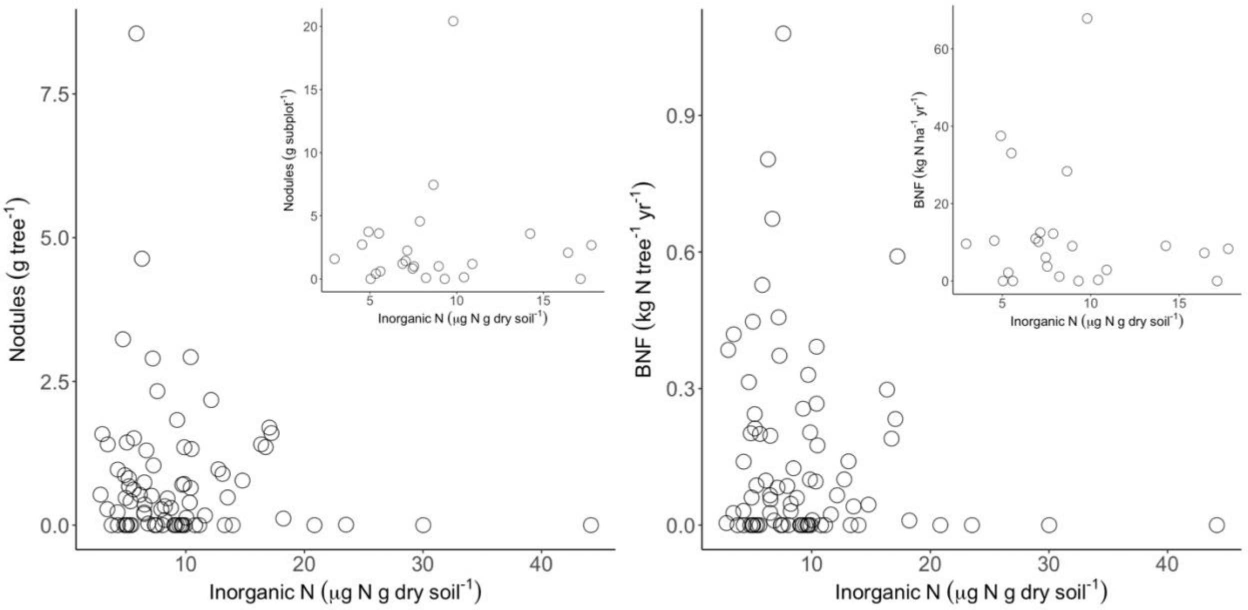


**Fig. S2.** Legume nodule mass and BNF versus soil inorganic N across individual stems in the 25 subplots within the VMFR. Insets show the relationship between nodule mass and BNF versus mean soil N at the subplot (20 x 20 m) scale. In all statistical analyses using linear mixed effects models and linear regression, we detected no significant ($P > 0.2$) effect of soil N on nodule mass or BNF.

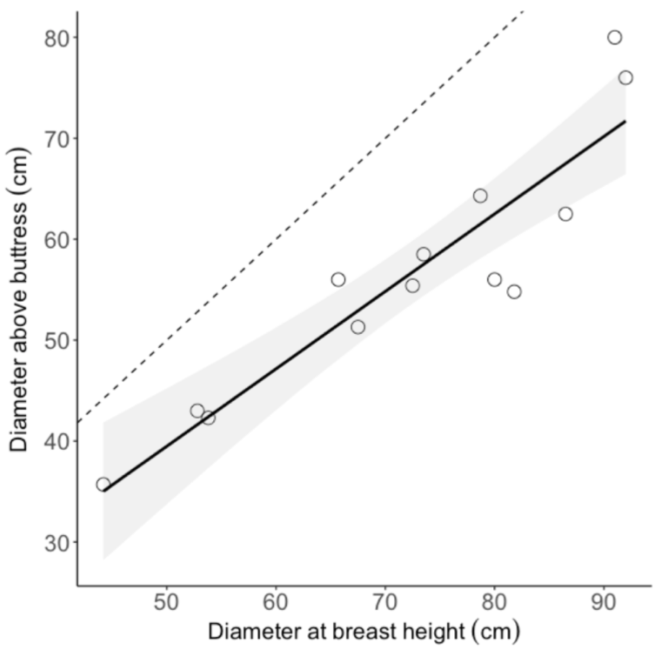


**Fig. S3.** Scaling relationship between tree diameter measured above the stem buttress vs. diameter measured at breast height (DBH). We used this empirical model $D_c = 0.767 * DBH + 1.115$ (linear regression; $R^2 = 0.85$, $P<0.001$) to correct diameters of trees that buttress at DBH for allometric calculations of biomass. The dashed line is the 1:1 line.

**Table S1.** Census intervals (number of censuses in parentheses), year of selective harvest (OG= Old-growth, never cut), long-term mean percent of stems that are *Pentaclethra macroloba* (PEMA) and long-term mean N fixer and non-fixer tree AGB among five 1-ha plots in VMFR.

| Plot | Interval | Harvest | PEMA (%) | N-fixer AGB (Mg $ha^{-1}$) | Non-fixer AGB (Mg $ha^{-1}$) |
|---|---|---|---|---|---|
| 5 | 1983-2013 (10) | 1981 | 21 | 56.7 | 236.4 |
| 18 | 1983-2013 (8) | 2000 | 45 | 96.6 | 137.6 |
| 26 | 1983-2009 (8) | 2005 | 26 | 68.2 | 111.4 |
| 30 | 1983-2002 (8) | 2012 | 34 | 85.9 | 128.0 |
| 41 | 1984-2009 (8) | OG | 14 | 46.6 | 265.6 |

**Table S2.** Summary table of Linear Mixed Effects model comparisons for AGB and stem demographics in our five 1-ha plots in the VMFR. Shown are ΔAICc values for each model tested with the best fit model shown in bold. Models for with ΔAICc < 2 are shown in italics, indicating that they yielded equivalent fits as the lowest AICc model.

| Response variable | Model | Δ AICc |
|---|---|---|
| AGB | Y ~ Type * Year, random =~1\| Plot | 140.80 |
| | **Y ~ Type * Year, random =~1\| Plot/Type** | **0** |
| | Y ~ Type * Year, random =~1\| Time | 151.66 |
| | Y ~ Type * Year, random =~1\| Time/Type | 154.05 |
| Stem RGR | Y ~ Type + Year, random =~1\| Plot | 2.37 |
| | **Y ~ Type + Year, random =~1\| Plot/Type** | **0** |
| | Y ~ Type + Year, random =~1\| Time | 12.59 |
| | Y ~ Type + Year, random =~1\| Time/Type | 10.31 |
| Stem Mortality | Y ~ Type + Year, random =~1\| Plot | 4.59 |
| | Y ~ Type + Year, random =~1\| Plot/Type | 6.96 |
| | **Y ~ Type + Year, random =~1\| Time** | **0** |
| | Y ~ Type + Year, random =~1\| Time/Type | 2.37 |
| AGB Growth | Y ~ Type + Year, random =~1\| Plot | 62.81 |
| | **Y ~ Type + Year, random =~1\| Plot/Type** | **0** |
| | Y ~ Type + Year, random =~1\| Time | 70.03 |
| | Y ~ Type + Year, random =~1\| Time/Type | 55.56 |
| AGB Mortality | *Y ~ Type + Year, random =~1\| Plot* | *1.22* |
| | Y ~ Type + Year, random =~1\| Plot/Type | 3.36 |
| | **Y ~ Type + Year, random =~1\| Time** | **0** |
| | Y ~ Type + Year, random =~1\| Time/Type | 2.16 |
| AGB Recruitment | Y ~ Type + Year, random =~1\| Plot | 2.21 |
| | **Y ~ Type + Year, random =~1\| Plot/Type** | **0** |
| | Y ~ Type + Year, random =~1\| Time | 2.72 |
| | Y ~ Type + Year, random =~1\| Time/Type | 2.37 |
| Net AGB Change | Y ~ Type + Year, random =~1\| Plot | 3.73 |
| | **Y ~ Type + Year, random =~1\| Plot/Type** | **0** |
| | Y ~ Type + Year, random =~1\| Time | 4.61 |
| | Y ~ Type + Year, random =~1\| Time/Type | 6.62 |

**Table S3.** Summary table of Linear Mixed Effects model comparisons for BNF across our 25 subplots in our five 1-ha plots in the VMFR. Shown are ΔAICc values for each model tested with the best fit model shown in bold. Models for with ΔAICc < 2 are shown in italics, indicating that they yielded equivalent fits as the lowest AICc model.

| Response variable | Model | Δ AICc |
|---|---|---|
| BNF | *Y ~ $AGB_{tot}$, random =~1\| Plot* | *1.05* |
| | *Y ~ $AGB_{non}$, random =~1\| Plot* | *1.30* |
| | Y ~ $AGB_{fix}$, random =~1\| Plot | 6.15 |
| | Y ~ $Growth_{tot}$, random =~1\| Plot | 5.97 |
| | **Y ~ $Growth_{non}$, random =~1\| Plot** | **0** |
| | Y ~ $Growth_{fix}$, random =~1\| Plot | 5.48 |
| | Y ~ $Net_{tot}$, random =~1\| Plot | 6.14 |
| | Y ~ $Net_{non}$, random =~1\| Plot | 3.56 |
| | Y ~ $Net_{fix}$, random =~1\| Plot | 5.62 |
| | Y ~ DIN, random =~1\| Plot | 5.43 |

**Table S4. Parameter ranges and random sampling for Monte Carlo simulations of N demand.**

| Parameter/Variable | Units | Mean value | Random sampling |
|---|---|---|---|
| [a]Fraction of AGB allocated to stems | Unitless | 0.98 | Uniform (0.97, 0.99) |
| [a]Fraction of AGB allocated to leaves | Unitless | 0.02 | Uniform (0.01, 0.03) |
| [b]C:N of N fixer stems | kg C kg $N^{-1}$ | 167 | Log-normal (85, 311) |
| [b]C:N of non-fixer stems | kg C kg $N^{-1}$ | 294 | Log-normal (92, 1361) |
| [c]C:N of live N fixer leaves | kg C kg $N^{-1}$ | 15.2 | Factorial (1.25) |
| [d]C:N of live non-fixer leaves | kg C kg $N^{-1}$ | 26.3 | Factorial (1.25) |

[a]Chave et al. 2008; [b]Martin et al. 2014; [c]Tully et al. 2013; [d]Epihov et al. 2017
